# Prevalence, correlates, and patterns of leaving firearms unattended in vehicles among US firearm owners

**DOI:** 10.1186/s40621-026-00688-z

**Published:** 2026-05-24

**Authors:** Alexander Testa, Jennifer Thompson, Chelsea Carriker, Dylan Jackson, Daniel Semenza, Jack Tsai

**Affiliations:** 1 Department of Management, Policy and Community Health, School of Public Health, University of Texas Health Science Center at Houston, 1200 Pressler Street, Houston, TX 77030 USA; 2 Department of Epidemiology, School of Public Health, University of Texas Health Science Center at Houston, Houston, TX USA; 3 Department of Health Promotion and Behavioral Sciences, School of Public Health, University of Texas Health Science Center at Houston, Houston, TX USA; 4 Department of Population, Family and Reproductive Health, Johns Hopkins Bloomberg School of Public Health, Baltimore, MD USA; 5 Department of Sociology, Anthropology, and Criminal Justice, Rutgers University, Camden, NJ USA; 6 Department of Urban-Global Public Health, School of Public Health, Rutgers University, Piscataway, NJ USA; 7 New Jersey Gun Violence Research Center, Rutgers University, Piscataway, NJ USA

**Keywords:** Firearms, Firearm theft, Firearm storage, Vehicles, Public Safety

## Abstract

**Background:**

Each year, hundreds of thousands of firearms are stolen, and vehicles are the leading source of firearm thefts in the United States. A precursor to these events is leaving a firearm in a vehicle unattended. Yet, the correlates and patterns of leaving firearms unattended in vehicles remain poorly understood. The purpose of this study was to examine the prevalence, correlates, and patterns of leaving firearms unattended in vehicles among US firearm owners.

**Methods:**

Using a cross-sectional survey of firearm-owning adults administered online by YouGov in February–March 2026 (*n* = 1,856), ordinal logistic regression examined correlates of leaving firearms in unattended vehicles, including sociodemographic, perceptions of safety, strength of state firearm policies, and geographic factors. Among those reporting vehicle firearm storage (*n* = 468), firearm type, storage location, and reasons for leaving firearms in vehicles were descriptively examined.

**Results:**

Overall, 73.2% of respondents reported never leaving a firearm in a vehicle unattended, 14.7% reported rarely, 5.7% reported sometimes, 3.2% reported most of the time, and 3.2% reported always. Male sex (*v*. female) (aOR = 1.48; 95% CI, 1.07–2.06), non-Hispanic Black race/ethnicity (*v*. non-Hispanic White) (aOR = 2.06; 95% CI, 1.17–3.61), prior firearm theft (*v*. no prior firearm theft) (aOR = 2.54; 95% CI, 1.60–4.03), higher perception of fear of being shot (aOR = 1.09, 95% CI = 1.03, 1.15), higher perceived likelihood of defensive gun use (1.08, 95% CI = 1.03, 1.14), conservative ideology (*v*. very liberal) (aOR = 2.46, 95% CI = 1.34, 4.52), and rural residence (*v*. metropolitan) (aOR = 2.21; 95% CI, 1.49–3.26) were associated with higher odds of leaving firearms in a vehicle more frequently. Among respondents who reported leaving firearms in vehicles unattended, handguns were the most common type of firearm left in vehicles (74.5%). Respondents most commonly reported leaving a firearm in the center console (24.6%) or the glovebox (16.7%); 19.7% used a designed for firearm storage. Top reasons for storing in a vehicle included legal carry restrictions, wanting a firearm nearby, and convenience.

**Conclusions:**

Future research, intervention development, and testing are needed to reduce the leaving of firearms in vehicles unattended.

## Introduction

Hundreds of thousands of firearms are stolen each year in the United States (US) [1–3] with parked vehicles emerging as a leading source of firearm thefts [4–7]. Estimates suggest that a firearm is stolen from a vehicle every 9 min, [4] and that by 2022, approximately 9% of all vehicle break-ins had resulted in the theft of a firearm [5]. A precursor to these thefts is that a firearm is left in an unattended vehicle (i.e., a vehicle with no occupant present). In response, efforts have been made to curtail the unattended storage of firearms in vehicles through policy and public awareness initiatives. For example, the Biden-Harris administration issued guidance in encouraging policies that mandate secure firearm storage in vehicles.[8] States have enacted laws governing leaving a firearm in a vehicle unattended [9]. Recent examples include Colorado enacting legislation requiring firearms to be stored in a locked container when left unattended in a vehicle, [10] and in Virginia, a proposed law in 2026 aims to make it a misdemeanor to leave a firearm visible in a vehicle parked on a public street [11]. Federal and local law enforcement, as well as auto insurance groups, have also launched efforts to raise awareness of this issue among the public [12–16].

Despite the growing attention toward the storage of firearms in the home, [17, 18, 19] research on leaving firearms in vehicles remains limited. Existing studies have largely focused on the prevalence of this behavior. Tucker et al.‘s study of 408 adult male firearm owners oversampled for prior suicidal ideation and found that 40% reported at least occasionally storing firearms in vehicles [20]. A nationally representative survey conducted in 2016 found that approximately 11% of US firearm owners reported storing at least one firearm in a vehicle at their residence [21]. A five-state survey conducted in 2022 investigating various locking devices that firearm owners use found that that 7.1% of respondents reported using an in-vehicle keyed/PIN/dial lock and 3.5% reported using an in-vehicle biometric lock [22]. Additionally, a national study of Black and American Indian/Alaska Native (AIAN) adults found that 10% of Black firearm owners and 13% of AIAN firearm owners reported “almost always” or “always” storing at least one firearm in a vehicle [23]. In a separate study using data from 9 US states found that approximately 27% of firearm owners reported storing a firearm in a vehicle [24].

Despite these studies’ contributions, several critical knowledge gaps remain. First, existing evidence relies on data with limited geographic coverage [22, 24] or targeted subgroups, [20, 23] which limits generalizability to firearm owners nationally. Second, there is limited information on key correlates associated with leaving a firearm in a vehicle, including factors associated with broader firearm storage behaviors, such as sociodemographic factors, [18, 19, 25, 26] perceptions of safety, [18, 25, 27] state firearm policy context, [25, 28] and geographic factors [18, 25, 29]. Third, no prior research has examined where, within a vehicle, individuals most commonly leave a firearm. Finally, there is a lack of research on the reasons why individuals leave firearms in vehicles.

To address these gaps, this study uses data from a recent national survey of US firearm owners to examine the prevalence, correlates, and patterns of leaving a firearm unattended in a vehicle.

## Data

Data are from a national survey of firearm-owning adults administered by YouGov in February-March 2026 called The Firearm Storage and Theft from Vehicles Study. YouGov maintains an opt-in online research panel of US adults recruited continuously and verified at registration through email validation, double opt-in, device fingerprinting, and multi-source geolocation checks designed to detect duplicate or fraudulent accounts. YouGov draws survey samples using a sample-matching methodology in which a target sample is first drawn from a synthetic frame derived from the American Community Survey and other authoritative population sources, and panelists are then matched to the target sample on demographic and other characteristics using a proximity-based algorithm. Eligibility for the present study was determined from existing panel profile data and confirmed at survey entry by an affirmative response to: “Do you currently own a functioning firearm? – Please do not include air, paintball, BB, pellet, or firearms that cannot fire when answering.”

Eligible panel members were invited to complete an approximately 12-minute online survey covering experiences with firearm storage and theft from vehicles. Respondents were compensated with YouGov points, which are redeemable for cash or prizes. YouGov invited 2,208 US adult gun owners. Among this sample, 1,860 respondents consented to take the survey. After removing missing data for non-response, the final analytic sample included 1,856 adult firearm owners. YouGov used the 2024 Current Employment Statistics survey as a reference population to define a target sample of US adults and generate post-stratification weights using proximity-based matching on gender, age, race, and education. The study protocol was approved by the University of Texas Health Science Center at Houston Committee on the Protection of Human Subjects (HSC-SPH-24–0345).

### Variables

All respondents were asked about the frequency of *firearms left in unattended vehicles.* The subset of respondents who report leaving firearms in unattended vehicles is asked about the type of firearm(s) left in unattended vehicles, reasons for leaving a firearm in an unattended vehicle, and the location in the vehicle where *a firearm is stored.* Details on the measurement and response options are provided in Appendix A.

Covariates include a host of sociodemographic factors, [18, 19, 25, 26] perceptions of safety, [18, 25, 27] state firearm policy context, [25, 28], and geographic factors [18, 25, 29] that have been found to relate to firearm storage patterns in the home, but have not been explicitly examined in regard to vehicles. Variables include age, biological sex, race and ethnicity, marital status, annual family income, presence of a child under 18 in the home, veteran status, length of firearm ownership, prior firearm theft from any location, prior violent victimization, fear of being shot, [25] defensive gun use perceptions, [30, 31] political ideology, strength of state firearm policy using the Giffords Law Center Gun Law Scorecard, [28, 32, 33, 34] region, and urbanicity. Details on the variable measurement and coding are provided in Appendix B.

### Analytic approach

First, the summary statistics for the analytic sample (*N* = 1,856) are presented. Next, we examine the frequency of vehicle firearm storage by estimating a multiple ordinal logistic regression model with standard errors clustered by state. The sample is then restricted to those who report leaving a firearm in an unattended vehicle at some point (*n* = 468; i.e., excluding those who indicated “never” storing a firearm in a vehicle), and we analyze the prevalence of responses pertaining to (a) the type of firearm(s) typically left in vehicles, (b) the location where firearms are most commonly left in vehicles, and (c) the method of firearm storage in a vehicle. All analyses used post-stratification weights and were conducted in Stata SE Version 18 [35]. A Brant test revealed that the proportional odds assumption was not violated (*p =*.616). Statistical significance was specified at 2-tailed *p* <.05. Data analysis was performed between March and April 2026.

## Results

Table 1 presents summary statistics for the analytic sample. Overall, 73.2% (*n* = 1,388) of respondents reported never leaving a firearm in a vehicle unattended, 14.7% (*n* = 273) reported rarely, 5.7% reported sometimes (*n* = 90), 3.2% reported most of the time (*n* = 51), and 3.2% reported always (*n* = 54). The sample is 55.1 years old on average (range 21–94), is 64.5% male (*n* = 1,177), and is 8.8% Hispanic (*n* = 144), 7.6% non-Hispanic Black (*n* = 112), 7.9% non-Hispanic Other (*n* = 133), and 75.8% non-Hispanic White (*n* = 1,467).

**Table 1:** Summary Statistics of The Firearm Storage and Theft from Vehicles Study (*N* = 1,856),

Variable	Weighted %/Mean	Unweighted *n*
**Frequency of Firearm Left in Vehicle Unattended**		
Never	73.2	1,388
Rarely	14.7	273
Sometimes	5.7	90
Most of the time	3.2	51
Always	3.2	54
**Age (years)**		
21–35	13.5	120
36–45	18.5	324
46–55	12.7	252
56–65	26.9	628
66–75	20.8	381
76+	7.6	151
**Sex**		
Female	35.5	679
Male	64.5	1,177
**Race/Ethnicity**		
Non-Hispanic White	75.8	1,467
Non-Hispanic Black	7.6	112
Hispanic	8.8	144
Non-Hispanic Other	7.9	133
**Marital status**		
Not married	35.7	661
Married or partnered	64.3	1,195
**Education**		
High school or less	31.6	498
Some college or 2-year degree	32.9	627
4-year degree	22.3	453
Post-graduate degree	13.3	278
**Family income**		
Under $30,000	14.2	255
$30,000–$59,999	22.6	420
$60,000–$99,999	25.8	462
$100,000 or more	30.7	587
Prefer not to say	6.7	132
**Child in home**		
No	76.6	1,489
Yes	23.4	367
**Veteran status**		
No	81.7	1,495
Yes	18.3	361
**Prior Firearm theft**		
No	90.3	1,680
Yes	9.7	176
**Length of firearm ownership**		
1–2 years	5.1	67
3–5 years	12.5	180
6–10 years	15.3	271
11–20 years	16.2	316
> 20 years	50.9	1,022
**Prior crime victimization**		
No	79.3	1,456
Yes	18.5	362
Prefer not to answer	2.2	38
**Fear of being shot (range 0–20)**	3.62	—
**Defensive gun use intentions (range 0–12)**	3.45	—
**Political ideology**		
Very liberal	6.8	130
Liberal	11.5	193
Moderate	27.9	502
Conservative	29.8	580
Very conservative	20.7	397
Not sure	3.4	54
**State gun law strength (Giffords score)**		
F	43.9	819
D	2.0	40
C	15.9	308
B	14.9	283
A	23.3	406
**Region**		
Northeast	10.9	207
Midwest	22.0	421
South	44.3	847
West	22.9	381
**Urbanicity**		
Metro	76.3	1,416
Micropolitan/Small Town	15.1	281
Rural	8.6	159

Table 2 presents the multiple ordinal logistic regression examining factors associated with leaving a firearm unattended in a vehicle. The results indicate that older respondents were significantly less likely to leave firearms in vehicles unattended more frequently compared to those 35 or younger, with significant findings emerging for those aged 56–65 (adjusted odds ratio [aOR] = 0.45, 95% CI = 0.26, 0.76), 66–75 (aOR = 0.34, 95% CI = 0.17, 0.66) and 76 and older (aOR = 0.16, 95% CI = 0.08, 0.32). Males have a higher odds of leaving firearm in vehicles unattended more frequently compared to females (aOR = 1.48, 95% CI = 1.07, 2.06), and non-Hispanic Black respondents were more likely to leave a firearm in vehicles unattended more frequently compared to non-Hispanic White respondents (aOR = 2.06, 95% CI = 1.17, 3.61).

**Table 2:** Ordered Logistic Regression of Factors Associated with Leaving a Firearm in a Vehicle Unattended From The Firearm Storage and Theft from Vehicles Study (*N* = 1,856)

Variable	aOR	95% CI
**Age**,** years (Ref: 21–35)**		
36–45	0.68	0.37, 1.24
46–55	0.58	0.31, 1.09
56–65	0.45^**^	0.26, 0.76
66–75	0.34^**^	0.17, 0.66
76+	0.16^***^	0.08, 0.32
**Sex (Ref: Female)**		
Male	1.48^*^	1.07, 2.06
**Race/Ethnicity (Ref: Non-Hispanic White)**		
Non-Hispanic Black	2.06^*^	1.17, 3.61
Hispanic	1.28	0.79, 2.08
Non-Hispanic Other	1.01	0.62, 1.65
**Marital Status (Ref: Not married)**		
Married or partnered	1.19	0.84, 1.69
**Education (Ref: High school or less)**		
Some college or 2-year degree	1.03	0.71, 1.48
4-year degree	0.94	0.68, 1.29
Post-graduate degree	0.81	0.56, 1.19
**Family Income (Ref: Under $30**,**000)**		
$30,000–$59,999	0.86	0.53, 1.41
$60,000–$99,999	1.00	0.57, 1.77
$100,000 or more	1.41	0.89, 2.23
Prefer not to say	1.34	0.60, 2.99
**Child in Home (Ref: No)**		
Yes	1.07	0.72, 1.59
**Veteran Status (Ref: No)**		
Yes	1.20	0.87, 1.64
**Prior Firearm Theft (Ref: No)**		
Yes	2.54^***^	1.60, 4.03
**Length of Firearm Ownership (Ref: 1–2 years)**		
3–5 years	0.96	0.37, 2.51
6–10 years	1.37	0.54, 3.52
11–20 years	1.47	0.65, 3.28
> 20 years	1.59	0.71, 3.57
**Prior Crime Victimization (Ref: No)**		
Yes	1.11	0.78, 1.57
Prefer not to answer	0.52	0.19, 1.40
**Safety Perceptions**		
Fear of being shot	1.09^**^	1.03, 1.15
Defensive gun use intentions	1.08^**^	1.03, 1.14
**Political Ideology (Ref: Very liberal)**		
Liberal	1.95	0.74, 5.14
Moderate	2.05^*^	1.10, 3.81
Conservative	3.04^**^	1.58, 5.87
Very conservative	2.46^**^	1.34, 4.52
Not sure	1.94	0.94, 4.00
**State Gun Law Strength — Giffords Score (Ref: F)**		
D	0.87	0.57, 1.33
C	0.84	0.62, 1.15
B	0.71	0.45, 1.10
A	0.68^*^	0.47, 0.97
**Region (Ref: Northeast)**		
Midwest	0.70	0.40, 1.21
South	1.01	0.57, 1.77
West	0.80	0.44, 1.47
**Urbanicity (Ref: Metro)**		
Micropolitan/Small Town	1.32^*^	1.01, 1.72
Rural	2.21^***^	1.49, 3.26

aOR = adjusted odds ratio. 95% CI = 95% confidence interval. Model estimated using ordered logistic regression with probability weights and standard errors clustered by state (k = 50). Reference categories shown in parentheses

^***^p <.001 ^**^p <.01 ^*^p <.05

Having previously had a firearm stolen from any location was associated with a higher odds of leaving a firearm in a vehicle more frequently, compared with never having a firearm stolen (aOR = 2.54, 95% CI = 1.60, 4.03). A supplemental analysis (results not shown) re-estimated this pattern using a question specifically identifying individuals who previously experienced the theft of a firearm from a vehicle (*n* = 51) and found a positive association with leaving firearms in a vehicle unattended more frequently compared to those who never had a firearm stolen from a vehicle (aOR = 3.41, 95% CI = 1.74, 6.66). Higher scores on the fear of being shot index (aOR = 1.09, 95% CI = 1.03, 1.15) and perceptions of the likelihood of future defensive gun use index (aOR = 1.08, 95% CI = 1.03, 1.14) were associated with an increased odds of leaving a firearm in a vehicle unattended more frequently. Compared to those who identify as ‘very liberal’, there was a higher odds of leaving a firearm in a vehicle unattended more frequently among those who identify as moderate (aOR = 2.05, 95% CI = 1.10, 3.81), conservative (aOR = 3.04, 95% CI = 1.58, 5.87), and very conservative (aOR = 2.46, 95% CI = 1.34, 4.52).

Living in a state with an “A” score on firearm policy restrictiveness (i.e., strongest state laws) was associated with a lower odds of leaving a firearm in a vehicle unattended more often compared to those living in states with an “F” score (aOR = 0.68, 95% CI = 0.47, 0.97). Finally, compared to those living in a metropolitan area, living in a micropolitan/small town (aOR = 1.32, 95% CI = 1.01, 1.72) or rural area (aOR = 2.21, 95% CI = 1.49–3.26) was associated with a higher odds of leaving a firearm unattended in a vehicle more frequently. As a sensitivity analysis, the results were re-examined using a multiple logistic regression model that differentiates between those who never leave a firearm in a vehicle and those who do at some point (see Appendix C). These results remained substantively similar to the findings using the ordinal regression model.

**Fig. 1:**
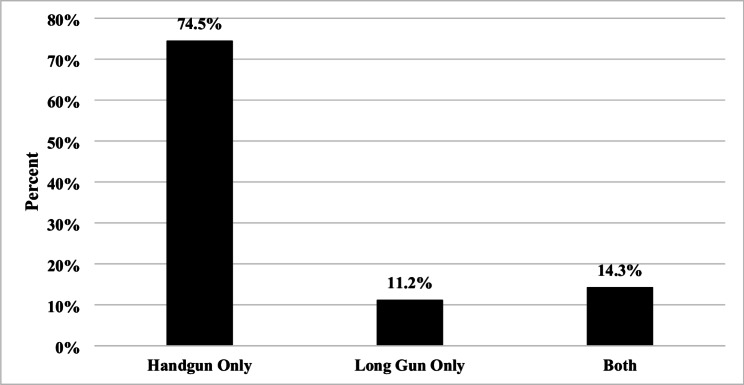
Type of Firearm Left Unattended in Vehicle From The Firearm Storage and Theft from Vehicles Study (*N* = 468)

Next, we restricted the sample to respondents who reported leaving a firearm in a vehicle at any point (*n* = 468). Figure 1 shows that 74.5% (*n* = 357) reported leaving only a handgun, 11.2% only a long gun (*n* = 49), and 14.3% both a handgun and a long gun (*n* = 62). Table 3 shows that the most commonly cited top-ranked reasons were legal restrictions on carrying a firearm in certain places (*n* = 162; 58.5%), wanting to have it nearby or readily available (*n* = 128; 43.8%), having a place in their vehicle where they can securely store firearms (*n* = 50; 28.1%), for convenience (*n* = 45; 22.7%), or that there was a law restricting the carrying of firearms in public (*n* = 27; 26.5%). Several individuals also provided an “other response,” in their top three ranking (*n* = 99) which was thematically categorized in Appendix D. Those responses most typically indicated that respondents reported not being able to bring their firearms into restricted or prohibited locations, that the firearm was left temporarily while in transit, for transportation during recreation or sporadic reasons, or because of personal safety when traveling.

**Table 3:** Top Three Reasons for Leaving a Firearm in a Vehicle Unattended From The Firearm Storage and Theft from Vehicles Study

Reason	Rank 1 *n* (%)	Rank 2 *n* (%)	Rank 3 *n* (%)	Total *N*
Legal restrictions on carrying firearms in certain places (e.g., workplace)	162 (58.5%)	74 (26.7%)	41 (14.8%)	277
I want to have a firearm nearby or readily available	128 (43.8%)	88 (30.1%)	76 (26.0%)	292
I have a place to securely store firearms in my vehicle	50 (28.1%)	75 (42.1%)	53 (29.8%)	178
For convenience (e.g., easier than carrying the firearm in and out)	45 (22.7%)	72 (36.4%)	81 (40.9%)	198
There is a law/ordinance restricting the carrying of firearms in public	27 (26.5%)	50 (49.0%)	25 (24.5%)	102
Lack of storage options outside of my vehicle (e.g., at work, at home)	18 (19.6%)	33 (35.9%)	41 (44.6%)	92
It is more convenient to leave my firearm in a vehicle than in my home	8 (17.0%)	14 (29.8%)	25 (53.2%)	47
I live with person(s) who do not want the firearm stored inside my residence	5 (26.3%)	6 (31.6%)	8 (42.1%)	19
It is something everyone in my community does	5 (9.8%)	24 (47.1%)	22 (43.1%)	51
Other	20 (20.2%)	18 (18.2%)	61 (61.6%)	99

Table is sorted by frequency of Rank 1 responses (descending) – with the exception of other response. Respondents selected up to three reasons and ranked them in order of importance (1 = most important)

**Fig. 2:**
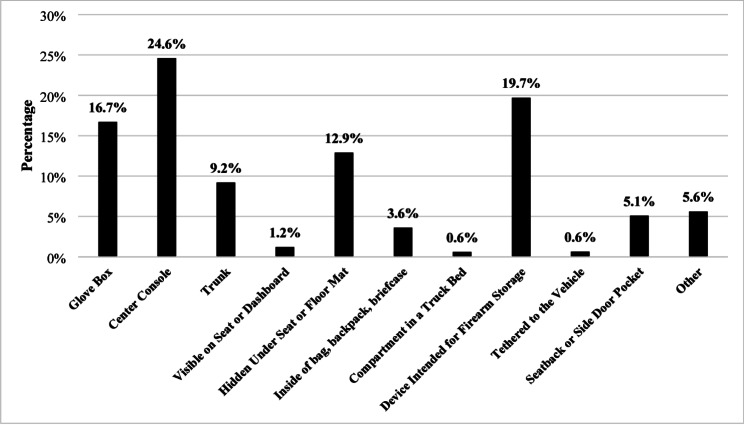
Method of Leaving a Firearm in a Vehicle Unattended From The Firearm Storage and Theft from Vehicles Study (*N* = 468)

Finally, Fig. 2 provides details on the firearm storage location. These results indicate that the most common locations were in the center console (24.6%; *n* = 115), a device intended for firearm storage (19.7%; *n* = 100), a glovebox (16.7%; *n* = 67), hidden under the seat or floor mat (12.9%; *n* = 55), or in the trunk (9.2%; *n* = 43). The other responses are thematically categorized in Appendix E (*n* = 28).

## Discussion

The current study offers several novel findings on leaving firearms in vehicles unattended. First, approximately 27% of firearm owners reported leaving a firearm unattended in a vehicle, consistent with prior research among firearm owners in nine states [24]. Additionally, roughly 6% reported doing so most of the time or always. Given that an estimated 31% of US adults own a firearm [36] (approximately 80.6 million individuals out of 260 million US adults [37], these prevalence estimates suggest that 21.8 million adults leave a firearm in a vehicle at some point, and approximately 4.8 million do so most of the time or always.

Next, the study examined factors associated with leaving a firearm unattended in a vehicle. Older respondents were less likely than younger respondents to leave a firearm unattended in a vehicle. In addition, male and non-Hispanic Black firearm owners had higher odds of leaving a firearm in a vehicle unattended. These demographic findings mirror research on correlates of unsecure firearm storage practices in the home [18]. These patterns suggest that outreach efforts aimed at promoting vehicle firearm storage messaging may benefit from targeting demographic groups at disproportionate risk of leaving a firearm in a vehicle unattended.

Individuals who had experienced a theft of a firearm from any location had over two times greater odds of currently storing a firearm in a vehicle unattended. These results are notable in that a prior theft does not appear to substantially alter risk behavior related to firearm storage in vehicles, and individuals who had a firearm previously stolen may be at elevated risk for a subsequent theft. There is a need for future research to further examine how prior theft influences current behavior of leaving firearms in vehicles, and whether shared risk factors unobserved in the current study underpin the relationship between prior firearm theft and current leaving of firearms in vehicles.

The findings also revealed that more conservative political ideology was associated with leaving a firearm in a vehicle more frequently. While limited research has investigated the role of political ideology in firearm storage behavior, these findings are consistent with prior work that has found more conservative political viewpoints are associated with storing firearms in a manner that enables more immediate firearm access in the home [25].

Fear of being shot and perceptions toward future defensive gun use were both associated with leaving a firearm in a vehicle unattended. Those who perceive a greater threat to their personal safety may feel a heightened need to have a firearm readily accessible at all times, including while outside of the home. These findings mirror prior research on threat perception that suggests concerns for personal safety are associated with the storage of firearms in the home, so that firearms are more readily accessible if needed for personal protection [25, 27, 38, 39]. This finding has important implications for intervention design, as messaging or policies aimed at reducing vehicle firearm storage may need to directly address firearm owners’ safety concerns and sense of vulnerability rather than focusing solely on the risks of theft of firearms from vehicles.

Residing in a state with more restrictive firearm laws was associated with a lower likelihood of leaving a firearm unattended in a vehicle. The impact of state-level firearm policies may operate through laws that directly restrict leaving firearms unattended in vehicles, limit public carrying, or mandate secure storage, as well as through norms around firearm carrying and storage behaviors. These results are consistent with research demonstrating that more restrictive state firearm policies are associated with secure firearm storage practices in the home [25, 28]. While several states have already enacted laws governing firearm storage in vehicles, [9, 10, 11] future research is needed to test the efficacy of such laws and whether policy changes influence leaving firearms in vehicles.

Those living in micropolitan/small towns or rural areas are more likely to leave firearms in vehicles compared to those in urban areas. Rural firearm owners may be more likely to routinely carry firearms for purposes such as hunting, wildlife encounters, or travel across large distances, making temporary vehicle storage a common part of daily routines. Additionally, rural areas may have fewer legal restrictions on firearm carrying and cultural norms that could increase the frequency with which firearms are both transported in and left in vehicles [18, 25, 29]. Findings suggest that geographically tailored interventions may be needed to account for the structural and cultural contexts that normalize leaving firearms in vehicles among rural firearm owners.

Firearm owners reported leaving handguns in vehicles more commonly than long guns [40]. This pattern may reflect that handguns are more commonly carried outside the home, particularly for personal protection, and are logistically more feasible to leave in a vehicle. Handguns being disproportionately left in vehicles unattended are consistent with prior research that found the majority of firearm thefts from vehicles result in a handgun being stolen [24]. Firearm owners most commonly store firearms in the center console, glove box, in a device intended for firearm storage, or under the seat. Notably, in most cases, the firearm is stored in a hidden location; however, these locations are also easily accessible in the event of a vehicle break-in. Future research is needed to understand firearm owners’ motivations for storing in specific locations, as well as the facilitators and barriers to using alternative storage methods, such as installing safes or firearm storage devices in vehicles.

Finally, the most common reasons for leaving firearms in vehicles included legal restrictions on carrying firearms in certain places, wanting a firearm nearby or readily available, having a place to store firearms in the vehicle, or convenience. The results create a notable juxtaposition: an unintended consequence of policies against firearms in public places may increase the likelihood of leaving firearms in vehicles. One consideration is that the enactment of these laws may be coupled with public outreach campaigns or efforts to promote more secure storage of firearms in vehicles, such as the use of vehicle safes to lock firearms. This study’s results point to the importance of coupling both public health messaging and policy efforts that aim to reduce firearms being left unattended in vehicles with efforts that address the safety concerns of firearm owners when they are outside the home.

## Limitations

There are limitations to the current study. First, because the study uses self-report survey data, the findings may be subject to social desirability bias about the frequency and manner in which individuals leave firearms in vehicles. Second, because the study uses cross-sectional data, the results should be interpreted as associations rather than causal, and not generalized beyond the single point in time during data collection from February-March 2026. Third, to the extent that leaving firearms in vehicles changes over the year, the findings may not reflect seasonal variation in firearm storage behaviors, as patterns of carrying and storing firearms may differ across seasons. Fourth, the measure of leaving firearms in vehicles unattended does not include details on certain characteristics, such as short-term compared to prolonged behavior, as well as how often the firearm is stored in secured (i.e., firearm lockbox or safe) compared to unsecured (i.e., on the seat or in the center console) locations. Fifth, it is important to note that the state-level policy measure from the Giffords Law Center Gun Law Scorecard is a broad, composite measure of state firearm laws and may not capture specific local or institutional policy context that may influence leaving firearms in vehicles unattended. Finally, it is important to note that due to the opt-in nature of the YouGov online panel, it is possible that firearm owners who choose to participate in online research panels may differ from non-participants in ways that correlate with leaving firearms in vehicles unattended. Future research should replicate these study findings using alternative sampling frames and recruitment methodologies.

## Conclusion

Leaving firearms in vehicles is a relatively common behavior, shaped by a combination of demographic factors, individual risk perceptions, contextual factors, and policy environments. Future research and intervention development aimed at reducing firearm storage in vehicles to reduce theft and injury should account for the motivations underlying this behavior, particularly perceived safety needs and structural constraints such as legal restrictions on carrying.

## Appendix A: Survey variables, questions, and response options from the firearm storage and theft from vehicles study

**Table:** 

Variable Label	Survey Question	Response Options
**Frequency of Firearm Left in Vehicle Unattended**	“How often do you leave your firearm(s) in a vehicle when there is no one in the vehicle?” Note: *Respondents were prompted that this refers to a firearm left without an adult present in the vehicle for any length of time.*	Never Rarely Sometimes Most of the time Always
*The following variables were assessed among the subset of respondents who answered more than “never” to the item above.*		
**Type of Firearm Left in Vehicle**	“What type of firearm do you most typically leave in your vehicle?”	Handgun only Long gun only Both
**Reasons for Leaving Firearm in a Vehicle**	Respondents were instructed to select and rank their top three reasons for leaving a firearm in a vehicle, indicating the most important reason as “1,” the second most important as “2,” and the third most important as “3.”	1. I have a place to securely store firearms in my vehicle 2. There are legal restrictions on carrying firearms in certain places (ex., workplaces, schools, hospitals, government buildings, or businesses that serve alcohol) 3. There is a law/ordinance restricting the carrying of firearms in public 4. It is more convenient to leave my firearm in a vehicle than in my home 5. Lack of storage options outside of my vehicle (ex., at work, at home, or in public spaces) 6. I want to have a firearm nearby or readily available 7. For convenience (ex., it is easier than carrying the firearm in and out) 8. It is something everyone in my community does 9. I live with a person/people who do not want the firearm stored inside my residence 10. Other (Please Specify)
**Firearm Storage Location in Vehicle**	“When you leave a firearm in a vehicle, how do you most often store it?”	Glove box Center console Trunk Visible on the seat or dashboard Hidden underneath the seat or floor mat Inside another item in the vehicle (e.g., backpack, bag, or purse) In a compartment in a truck bed In a device intended for firearm storage (e.g., lockbox or console safe) Tethered to the vehicle In a seatback or side door pocket Other (Please Specify)

## Appendix B. Study covariates, variable descriptions, and response options from the firearm storage and theft from vehicles study

**Table:** 

Variable Label	Variable Description/Survey Question	Response Options/Coding
***Sociodemographic Covariates (provided by YouGov)***		
**Age**	Continuous age in years provided by YouGov.	21–35 36–45 46–55 56–65 66–75 76+
**Sex**	Biological sex provided by YouGov.	Female Male
**Race/Ethnicity**	Race and ethnicity provided by YouGov.	Non-Hispanic White Non-Hispanic Black Hispanic Non-Hispanic other race
**Educational Attainment**	Highest level of education completed, provided by YouGov.	Less than college Some college College graduate Postgraduate
**Marital Status**	Current marital status provided by YouGov.	Currently married or partnered Not married or partnered
**Annual Family Income**	Annual family income provided by YouGov.	Less than $30,000 $30,000–$59,999 $60,000–$99,999 $100,000 or more Prefer not to say
**Child Under 18 in Home**	Presence of a child under 18 years of age in the household, provided by YouGov.	Yes No
**Veteran Status**	Having previously served in the US military, provided by YouGov.	Yes No
***Firearm-Related Covariates***		
**Length of Firearm Ownership**	“In total, how many years have you owned any type of firearm(s)?”	Less than 3 years 3–5 years 6–10 years 11–20 years More than 20 years
**Prior Firearm Theft from Any Location**	“Have you ever had any of your firearms stolen from any location?”	Yes No
***Violence Experience and Safety Perceptions***		
**Prior Violent Victimization**	“Have you ever been a victim of a crime where you were physically injured with violence?”	No Yes Prefer not to answer
**Fear of Being Shot**	“How worried are you about being shot: (1) in your home, (2) in your neighborhood, (3) in public (ex., grocery store, movie theater, restaurant), (4) at school, (5) at work, and (6) by the police. Items were summed to create a composite firearm violence worry scale (range: 0–20), with higher scores indicating greater concern. (Cronbach’s α =.821)	Each item rated on a 4-point Likert scale: 0 = Not worried at all 1 = Slightly worried 2 = Moderately worried 3 = Very worried Composite score range: 0–20
**Defensive Gun Use Intentions**	“Thinking about the future, how likely are you to do any of the following behaviors with a firearm to protect yourself or another person?:” (1) Tell someone who is threatening them that you have a firearm; (2) show your firearm to someone who is threatening them; (3) fire your firearm in the vicinity of but not at someone who is threatening them; and (4) fire your firearm at someone who is threatening them. Items were summed to create a composite defensive gun use intentions scale (range: 0–12), with higher scores indicating greater intention to use a firearm defensively. (Cronbach’s α = 0.744)	Each item rated on a 4-point Likert scale: 0 = Not at all likely 1 = Slightly likely 2 = Moderately likely 3 = Extremely likely Composite score range: 0–12
***Political and Contextual Variables***		
**Political Ideology**	“In general, how would you describe your own political viewpoint?”, provided by YouGov.	Very liberal Liberal Moderate Conservative Very conservative Not sure
**State Firearm Policy Restrictiveness**	Measured using the Giffords Law Center Gun Law Scorecard, which ranks states based on the strength of their firearm laws. States are assigned a grade ranging from “A” to “F,” with higher scores indicating more restrictive policy environments.	State grade: A to F Higher scores = more restrictive
**Region**	Geographic region of residence based on the respondent’s zip code, maintained by YouGov.	Northeast Midwest South West
**Urbanicity**	Level of urbanization of the respondent’s area of residence based on RUCA codes on the zip code maintained by YouGov.	Rural Micropolitan/small town Metropolitan

## Appendix C: Multiple logistic regression of factors associated with leaving a firearm in a vehicle unattended. from the firearm storage and theft from vehicles study (*N* = 1,856)

**Table:** 

Variable	AOR	95% CI
Age, years (Ref: 21–35)		
36–45	0.72	0.41, 1.25
46–55	0.53^*^	0.29, 0.96
56–65	0.44^**^	0.27, 0.72
66–75	0.32^***^	0.17, 0.60
76+	0.16^***^	0.08, 0.29
**Sex (Ref: Female)**		
Male	1.43^*^	1.03, 1.98
**Race/Ethnicity (Ref: Non-Hispanic White)**		
Non-Hispanic Black	2.56^**^	1.47, 4.43
Hispanic	1.43	0.92, 2.22
Non-Hispanic Other	1.00	0.60, 1.67
**Marital Status (Ref: Not married)**		
Married or partnered	1.15	0.80, 1.65
**Education (Ref: High school or less)**		
Some college or 2-year degree	0.91	0.64, 1.29
4-year degree	0.91	0.66, 1.26
Post-graduate degree	0.73	0.50, 1.08
**Family Income (Ref: Under $30**,**000)**		
$30,000–$59,999	1.06	0.65, 1.74
$60,000–$99,999	1.28	0.73, 2.23
$100,000 or more	1.70^*^	1.04, 2.79
Prefer not to say	1.49	0.68, 3.26
**Child in Home (Ref: No)**		
Yes	0.95	0.67, 1.34
**Veteran Status (Ref: No)**		
Yes	1.26	0.89, 1.78
**Prior Firearm Theft (Ref: No)**		
Yes	2.22^**^	1.40, 3.51
**Length of Firearm Ownership (Ref: 1–2 years)**		
3–5 years	1.34	0.56, 3.21
6–10 years	1.80	0.79, 4.08
11–20 years	2.02	0.97, 4.22
> 20 years	2.23^*^	1.05, 4.73
**Prior Crime Victimization (Ref: No)**		
Yes	1.17	0.83, 1.65
Prefer not to answer	0.59	0.21, 1.69
**Continuous Predictors**		
Fear of crime scale	1.07^*^	1.01, 1.13
Defensive gun use scale	1.07^**^	1.03, 1.12
**Political Ideology (Ref: Very liberal)**		
Liberal	1.83	0.71, 4.73
Moderate	2.14^*^	1.12, 4.08
Conservative	3.24^***^	1.69, 6.20
Very conservative	2.66^**^	1.48, 4.79
Not sure	2.00	0.88, 4.54
**State Gun Law Strength — Giffords Score (Ref: F)**		
D	1.20	0.76, 1.88
C	0.88	0.68, 1.14
B	0.74	0.47, 1.19
A	0.62^*^	0.42, 0.91
**Region (Ref: Northeast)**		
Midwest	0.70	0.42, 1.19
South	1.01	0.60, 1.70
West	0.89	0.51, 1.54
**Urbanicity (Ref: Metro)**		
Micropolitan/Small Town	1.46^*^	1.07, 2.00
Rural	1.97^**^	1.34, 2.90

AOR = adjusted odds ratio. 95% CI = 95% confidence interval. Model estimated using binary logistic regression with probability weights and standard errors clustered by state (k = 50). Reference categories shown in parentheses

^***^p <.001 ^**^p <.01 ^*^p <.05

## Appendix D: Thematic categorization of open-ended responses: reasons for leaving firearms in unattended vehicles from the firearm storage and theft from vehicles study (*N* = 99)

**Table:** 

Theme	*n*	Example Responses
**Legal or Policy Restriction on Carrying in Establishment**	19	“I do not bring the weapon inside the restaurant”; “can’t carry my rifle into church when we will be going to the range later”; “if the place I’m going (i.e. banks, etc) into doesn’t allow them”; “only when I go to hospital”; “workplace doesn’t allow on property”; “Can’t take it in the place I am going”
**Personal Safety or Self-Protection While Traveling**	16	“For safety when traveling to unknown areas”; “Feel safe with it in there”; “When I’m traveling out of state”; “Safety when I travel alone”; “Personal safety”; “For safety in case of people try to hurt me or rob me while outside of my home”; “Potential terror or mass shooting event”
**Survey Response Inadequacy or No Valid Answer**	14	“I would have picked my 1 st choice again”; “There were no other choices”; “Needed a reason”; “No other answer seemed appropriate”; “There isn’t any other reason, just the 2 I replied”
**Recreation or Transport to Range or Hunting**	14	“Transporting to a place to hunt camp or fish or target shoot”; “Transit to shooting areas”; When going hunting or going to range”; “I keep it in my car when use it at the range”; “Hunting big game”; “Taking it to the range”;
**Brief or Temporary Stop**	13	“Brief trip into a convenience store”; “Fueling my car”; “It’s only for 5 min”; “I’m in transit and need to stop (like for gas, or to run into a shop briefly)”
**Concealment or Discretion Concerns**	6	“To conceal from public view”; “It is more easily concealed inside the vehicle”; “Can’t always carry hidden if not wearing attire that allows that”; “Hang to have without everyone knowing where it is”
**Discretionary Carrying/Storage**	6	“Because I can”; “I am in an area where I will not need it”; “Don’t need/want to carry it”; “I want to”; “Don’t feel like carry on my body”
**Does Not Leave Firearm in Vehicle**	4	“I would never leave my gun in a car”; “I never leave a firearm in my vehicle”; “I do not leave my firearm unattended in my vehicle”
**Forgetting or Inadvertent Storage**	3	“Forget sometimes”; “Forget to remove it”; “Forget to take it inside”
**Rural Area**	3	“For ease of ease in my rural areas”; “In case we accidentally hit or find a wounded animal”; “Work nights and live in a rural area”.
**Secure Storage Device in Vehicle**	1	“It’s locked in a box”;

## Appendix E: Thematic categorization of open-ended responses: locations where firearms are left in vehicles from the firearm storage and theft from vehicles study (*N* = 28)

**Table:** 

Theme	*n*	Example Responses
**Refusal to Answer/Does Not Apply**	11	“Not saying”; “Nice try”; “I do not feel comfortable answering this question”; “Why do you want to know?”
**Hidden/Out of Sight**	4	“Outside of line of sight”; “Hidden”; “It’s black and the carpet is black so it’s hard to see and find”; “Hidden under something”
**Dedicated Storage Device or Case**	4	“In a tethered device intended for firearm storage”; “Safe”; “In a case under other luggage”; “In back of UV out of site and locked in a case”
**Behind or Under Seat**	3	“Behind my truck seat”; “Behind the truck seat”; “Backseat floor”;
**Beside/Adjacent to Seat**	3	“Beside the seat”; “Between seat and sole”; “Behind door”
**Hidden Compartment**	2	“Under backseat in a compartment”; “Hidden compartment in floorboard of my pickup”
**Carried on Person**	1	“On my person”

## Data Availability

No datasets were generated or analysed during the current study.
